# Identification and clinical value of a new ceRNA axis (TIMP3/hsa‐miR‐181b‐5p/PAX8‐AS1) in thyroid cancer

**DOI:** 10.1002/hsr2.1859

**Published:** 2024-02-25

**Authors:** Jiamin Liang, Yu Deng, Yubi Zhang, Bin Wu, Jing Zhou

**Affiliations:** ^1^ Department of Breast and Thyroid Surgery, Union Hospital, Tongji Medical College Huazhong University of Science and Technology Wuhan China; ^2^ Department of Orthopaedics, Union Hospital, Tongji Medical College Huazhong University of Science and Technology Wuhan China; ^3^ Department of Breast and Thyroid Surgery, People's Hospital of Dongxihu District Wuhan City and Union Dongxihu Hospital Huazhong University of Science and Technology Wuhan China

**Keywords:** ceRNA, hsa‐miR‐181b‐5p, PAX8‐AS1, TC, TIMP3, tumor immunity

## Abstract

**Background:**

Thyroid cancer (TC) is a prevalent and increasingly common malignant tumor. In most cases, TC progresses slowly and runs a virtually benign course. However, challenges remain with the treatment of refractory TC, which does not respond to traditional management or is subject to relapse or metastasis. Therefore, new therapeutic regimens for TC patients with poor outcomes are urgently needed.

**Methods:**

The differentially expressed RNAs were identified from the expression profile data of RNA from TC downloaded from The Cancer Genome Atlas database. Multiple databases were utilized to investigate the regulatory relationship among RNAs. Subsequently, a competitive endogenous RNA (ceRNA) network was established to elucidate the ceRNA axis that is responsible for the clinical prognosis of TC. To understand the potential mechanism of ceRNA axis in TC, location analysis, functional enrichment analysis, and immune‐related analysis were conducted.

**Results:**

A ceRNA network of TC was constructed, and the TIMP3/hsa‐miR‐181b‐5p/PAX8‐AS1 ceRNA axis associated with the prognosis of TC was successfully identified. Our results showed that the axis might influence the prognosis of TC through its regulation of regulating tumor immunity.

**Conclusions:**

Our findings provide evidence that TIMP3/hsa‐miR‐181b‐5p/PAX8‐AS1 axis is significantly related to the prognosis of TC. The molecules involved in this axis may serve as novel therapeutic approaches for TC treatment.

## 1 INTRODUCTION

Thyroid cancer (TC), a common malignancy in the endocrine system, is a solid tumor. Its incidence has been rapidly rising worldwide in recent years. From 2003 to 2011, the annual growth rate of TC in Chinese women was as high as 20.1%.^1^ While the vast majority of TC cases progress slowly, following a benign course, and being curable through standard surgery, postoperative radioactive I^131^ therapy, and thyroid stimulating hormone suppression, some types of TC (such as refractory differentiated TC of I^131^ and low‐undifferentiated TC) are unresponsive to I^131^ therapy and traditional chemotherapy. These types are prone to recur and metastasize, resulting in poor outcomes.^2^, ^3^ The refractory TC poses a great challenge to clinicians and has been a major contributor to a 1.6% annual increase in the death rate of TC in China over the past decade.^1^ Recent advancements in the realm of molecular targeted drugs have led to a significant improvement in the treatment of refractory TC.^4^, ^5^ The mitogen‐activated protein kinase (MAPK) pathway is considered vital in the development and progression of TC, and targeting BRAF mutation, RET/PTC gene rearrangement, and RAS mutation has demonstrated promise in TC treatment.^6^ Based on these discoveries, scientists have successfully developed drugs such as the BRAFV600E inhibitor, mammalian target of rapamycin inhibitor, and antiangiogenesis tyrosine kinase inhibitor, which have improved the quality of life for patients with refractory TC.^2^, ^3^, ^7^ However, despite the potential advantages of molecular targeted medications, several challenges persist, including drug resistance, recurrence, adverse reactions, and even disease deterioration.^8^, ^9^, ^10^ To overcome these challenges, researchers are endeavoring to investigate the mechanisms of TC to identify new therapeutic targets for the treatment of refractory TC and improve patient prognosis.

In recent studies, it has been discovered that messenger RNA (mRNA), microRNA (miRNA), and long noncoding RNA (lncRNA) interact with each other, resulting in the formation of a competitive endogenous RNA (ceRNA) regulatory network.^11^, ^12^ miRNA has the ability to bind to specific mRNA, leading to its silencing, and negatively regulate the stability or translation process of its targeted mRNA, playing a crucial role in various cellular processes, including proliferation, apoptosis, and growth.^13^ A reciprocal regulation has been identified between lncRNA and miRNA. LncRNA possesses the capacity to competitively interact with miRNA, leading to a decrease in the concentration of the targeted miRNA. This phenomenon, in turn, indirectly hinders the interaction between mRNA and commonly occurring miRNA response elements, thereby upregulating the expression of mRNA.^14^, ^15^ Additionally, it is important to highlight that a considerable proportion of lncRNA shares structural resemblances with mRNA, suggesting that miRNA may have a suppressive impact on the expression of lncRNA through a mechanism similar to what is observed with mRNA. Multiple studies have presented compelling evidence that supports the participation of this regulatory mechanism in a range of malignancies, such as pancreatic cancer, hepatocellular carcinoma, breast cancer, and TC. It has been demonstrated that it plays a significant role in the regulation of tumor proliferation and metastasis.^16^, ^17^, ^18^, ^19^ However, the exact role of the ceRNA network in the pathogenesis of TC has not been fully comprehended. Given the rising mortality rate of TC, it is of utmost importance to identify and delineate a pivotal ceRNA network that exhibits a significant association with TC to improve the prognosis of this disease.

**Figure 1:**
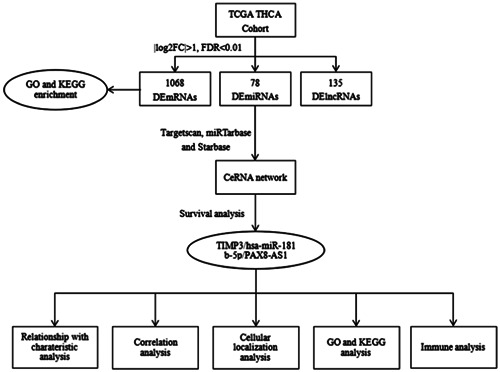
Flowchart of construction and analysis of ceRNA. ceRNA, competitive endogenous RNA; deRNA; differentially expressed RNA; FC, fold change; FDR, false discovery rate; GO, gene ontology; KEGG, Kyoto Encyclopedia of Genes and Genomes; lncRNA, long noncoding RNA; miRNA, microRNA; TCGA‐THCA, The Cancer Genome Atlas Thyroid Cancer.

In this study, a ceRNA network was constructed in TC. First, a TC‐related ceRNA network was established by selecting differentially expressed RNAs (deRNAs) between TC and normal thyroid tissues. A survival analysis was conducted to assess the hub RNAs within the network. Then, the ceRNA axis (TIMP3/hsa‐miR‐181b‐5p/PAX8‐AS1), which is significantly related to prognosis, was successfully identified. Subsequently, lncRNA localization analysis, enrichment analysis, and immune analysis were performed on this ceRNA axis to investigate its potential mechanism in TC, with an attempt to identify a new target for the treatment of TC and enhance the prognosis of patients with refractory TC (Figure 1).

## 2 MATERIALS AND METHODS

### 2.1 Data collection

Clinical information and corresponding RNA data of TC samples were acquired from The Cancer Genome Atlas (TCGA) data portal (version 28.0, accessed on March 22, 2021), and the RNA and miRNA sequence data type used was gene expression quantification‐HTseq‐Counts. All data could be freely available for download (https://portal.gdc.cancer.gov/).^20^ This study was exempted from the approval by the local ethics committee, and adhered to the data access policies and publication guidelines set forth by TCGA.

### 2.2 Identification of deRNAs

The EdgeR package in R software (version 3.5.1) was employed to perform normalization and further processing of the RNA data. DeRNAs between TC and normal tissues were detected using the Limma package. All *p* values were corrected for multiple comparisons using the false discovery rate (FDR). The selection of deRNAs was based on the following criteria: FDR < 0.01 and |log_2_ fold change (FC)| > 1. The FC represents the ratio of expression values between the two groups of samples. Volcano maps were generated using the gplots package for eligible deRNAs that were identified.

### 2.3 Functional enrichment analysis

To comprehensively examine the functional characteristics of deRNAs, gene ontology (GO) analysis and Kyoto Encyclopedia of Genes and Genomes (KEGG) pathway enrichment analysis were performed using the clusterprofiler package and org.Hs.eg.db package in R software. The GO analysis encompassed the examination of biological processes, cellular components, and molecular functions. The cut‐off values for both GO analysis and KEGG pathway analysis were set at a *p* value < 0.01 and a *q* value < 0.01. Additionally, the DAVID database (https://david.ncifcrf.gov/tools.jsp, accessed on June 22, 2022) was also utilized for functional enrichment analysis of mRNA.^21^

### 2.4 Construction of ceRNA network

The construction of ceRNA network of TC involved the following steps. Initially, the R software programming language was used to filter interactions among deRNAs from the miRcode database. Then, the Targetscan (https://www.targetscan.org/vert_71/),^22^ miRTtarbase (https://mirtarbase.cuhk.edu.cn/),^23^ and Starbase (https://starbase.sysu.edu.cn/index.php)^24^ databases were searched for the targeted mRNAs of demiRNAs. Subsequently, the Starbase database was employed to predict the relationship between lncRNAs and demiRNAs. To enhance the robustness of our research, only interactions covered by all three databases were included in the ceRNA network construction. Finally, the Cytoscape software (version 3.8.2) was utilized to build the mRNA‐miRNA‐lncRNA regulatory network.

### 2.5 Survival analysis

The Survival package of R software was utilized to perform survival analysis on all deRNAs. To validate the results of the survival analysis of mRNA, miRNA, and lncRNA, the GEPIA2 database (http://gepia2.cancer-pku.cn/#index, accessed on June 13, 2022) and the CancerMIRNome database(http://bioinfo.jialab-ucr.org/CancerMIRNome/, accessed on June 13, 2022)^25^ were employed. A *p* value below 0.05 was considered to be statistically significant.

### 2.6 Prediction of binding sites

The binding sites of hsa‐miR‐181b‐5p with TIMP3 and PAX8‐AS1 were forecasted by employing the Starbase database (accessed on June 13, 2022).

### 2.7 Intracellular localization of lncRNA

The sequence information of the lncRNA was obtained from the University of California Santa Cruz (UCSC) website (https://genome.ucsc.edu/cgi-bin/hgTracks, accessed on June 17, 2022).^26^ To determine its subcellular localization, lncLocator (http://www.csbio.sjtu.edu.cn/bioinf/lncLocator/)^27^ was applied.

### 2.8 Tumor immunity correlation analysis

To evaluate the relationship between TIMP3 and TC immunity, the correlation of immune cell infiltration, immunomodulators, major histocompatibility complex (MHC) molecules, chemokines, and their receptors were analyzed. To achieve this, the TIMER database (https://cistrome.shinyapps.io/timer/, accessed on June 25, 2022)^28^ and the TISIDB database (http://cis.hku.hk/TISIDB/, accessed on June 25, 2022)^29^ were employed.

### 2.9 Statistical analysis

All data were analyzed using R version 3.5.1, and all experiments were performed in triplicate. Kaplan–Meier analysis was performed using the “Survival” package in R to evaluate the survival outcomes of groups classified as high risk and low risk. A *p* value less than 0.05 indicated statistical significance.

## 3 RESULTS

### 3.1 DeRNAs in TC and normal tissues

**Figure 2:**
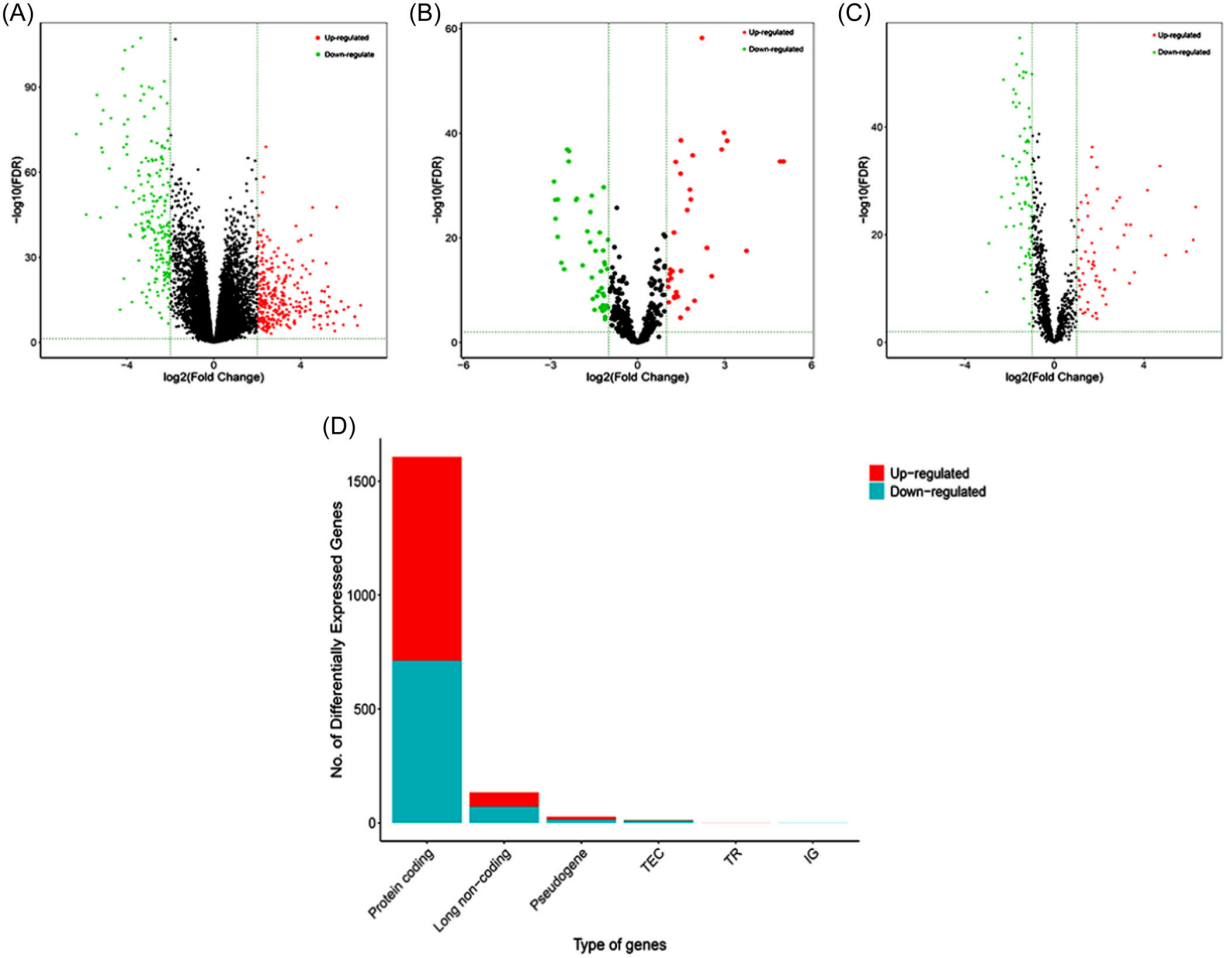
Volcano plots and stacked bar chart of deRNAs comparing TC and normal tissues. Red represents upregulated genes and green denotes downregulated genes. The volcano plots describe (A) 1068 demRNAs, (B) 78 demiRNAs, and (C) 135 delncRNAs. (D) The stacked bar chart shows the distribution of DEGs among different gene types. DEGs, differentially expressed genes; deRNA; differentially expressed RNA; lncRNA, long noncoding RNA; mRNA, messenger RNA; miRNA, micro RNA; TC, thyroid cancer.

The mRNA and lncRNA sequences of 510 TC samples and 58 normal samples, along with the miRNA sequences of 514 TC samples and 59 normal samples were obtained from TCGA database. A total of 1608 differentially expressed mRNAs (demRNAs) were identified, with 896 showing upregulation and 712 showing downregulation. Additionally, 78 differentially expressed miRNAs (demiRNAs) were observed, with 33 presenting upregulation and 45 downregulation. Furthermore, 135 differentially expressed lncRNAs (delncRNAs) were found, with 64 being upregulated and 71 downregulated, in both TC and normal tissues. Finally, the distribution of these deRNAs was displayed with volcano plots and stacked bar charts (Figure 2).

### 3.2 GO function enrichment analysis and KEGG pathway analysis

**Figure 3:**
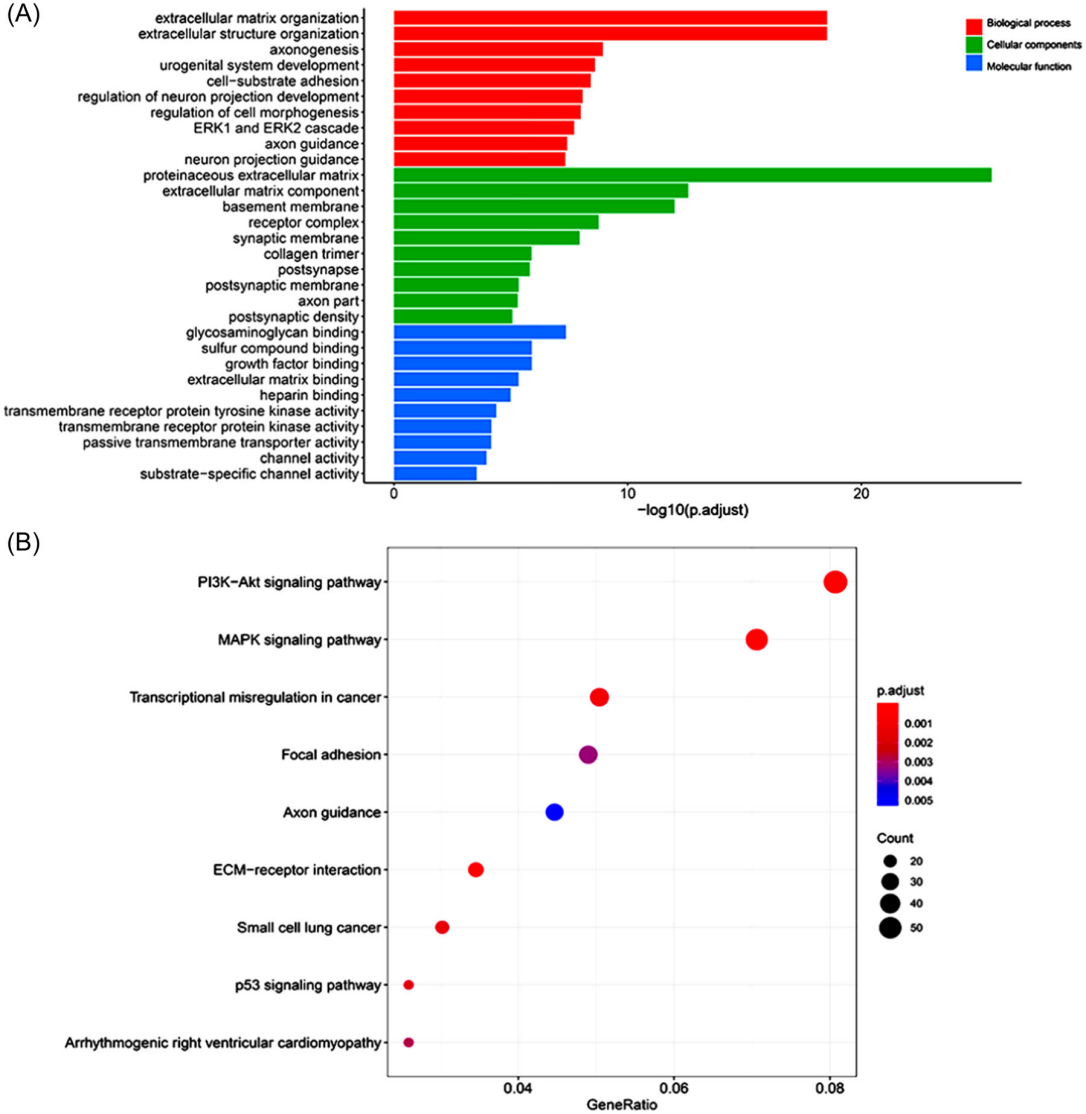
Functional enrichment analysis of demRNAs. (A) The enrichment scores of top 10 significantly regulated terms of biological process, cellular components, and molecular function. (B) KEGG enrichment of demRNAs. demRNAs; differentially expressed messenger RNAs; ECM, extracellular matrix; KEGG, Kyoto Encyclopedia of Genes and Genomes; MAPK, mitogen‐activated protein kinase.

The results of GO enrichment analysis of DEGs are shown in Figure 3. The most significant biological processes identified in this study include extracellular matrix (ECM) organization, extracellular structure organization, and axonogenesis. As for molecular functions, the notable terms are glycosaminoglycan binding, sulfur compound binding, and growth factor binding. Considering cellular components, the most common terms are proteinaceous ECM, ECM component, and basement membrane (Figure 3A). Figure 3B illustrates the results of KEGG pathway analysis. The DEGs were significantly enriched in the ECM–receptor interaction, MAPK signaling pathway, and PI3K‐Akt signaling pathway in TC (*p* < 0.01).

### 3.3 Construction and analysis of ceRNA network

**Figure 4:**
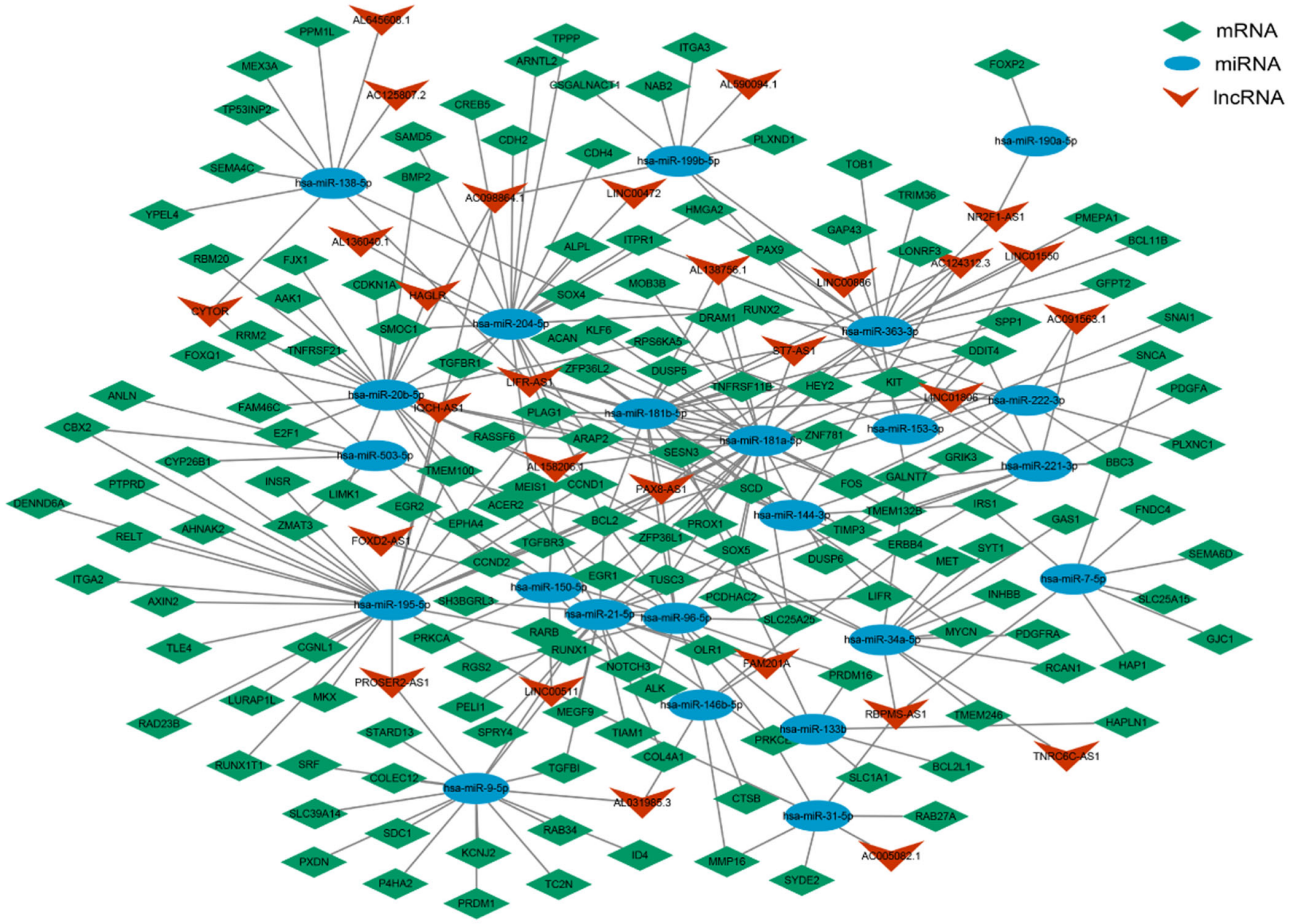
ceRNA regulatory network of thyroid cancer (diamonds are mRNAs, ovals represent miRNAs, and arrows denote lncRNAs). ceRNA, competitive endogenous RNA; mRNAs, messenger RNAs; miRNAs, micro RNAs; lncRNAs, long noncoding RNAs.

To further investigate the potential interaction between deRNAs, a combination of miRcode, TargetScan, miRTarbase, and Starbase databases were applied to predict the targeted regulatory relationship between deRNAs. Finally, this analysis identified a total of 145 mRNAs, with 78 being upregulated and 67 downregulated. Additionally, 22 miRNAs were identified, with 10 exhibiting upregulation and 12 downregulation. Furthermore, 28 lncRNAs were identified, with 10 displaying upregulation and 18 downregulation (Figure 4). The degree of RNA represents the closeness of nodes in the network to other RNAs, and nodes with higher degrees are generally considered to have greater importance in the overall connectivity of the network. Therefore, the core nodes were identified by analyzing the degree of RNAs in the network, and the top 20 nodes were found to play a greater role in the ceRNA network. Notably, hsa‐miR‐181a‐5p was found to regulate most DEGs, while BCL2 was targeted by a large number of miRNAs (Table 1).

**Table 1:** Top 20 nodes in the network according to degree.

Rank	Name	Score	Rank	Name	Score
1	hsa‐miR‐181a‐5p	33	11	hsa‐miR‐221‐3p	10
2	hsa‐miR‐195‐5p	28	12	hsa‐miR‐138‐5p	9
3	hsa‐miR‐20b‐5p	22	12	hsa‐miR‐144‐3p	9
4	hsa‐miR‐181b‐5p	21	12	BCL2	9
5	hsa‐miR‐204‐5p	20	15	hsa‐miR‐222‐3p	8
6	hsa‐miR‐363‐3p	18	15	hsa‐miR‐7‐5p	8
7	hsa‐miR‐9‐5p	17	17	hsa‐miR‐153‐3p	7
8	hsa‐miR‐21‐5p	15	17	hsa‐miR‐199b‐5p	7
9	hsa‐miR‐34a‐5p	13	17	hsa‐miR‐31‐5p	7
9	hsa‐miR‐96‐5p	13	17	hsa‐miR‐503‐5p	7

### 3.4 Selection of hub RNAs in light of prognostic values

**Figure 5:**
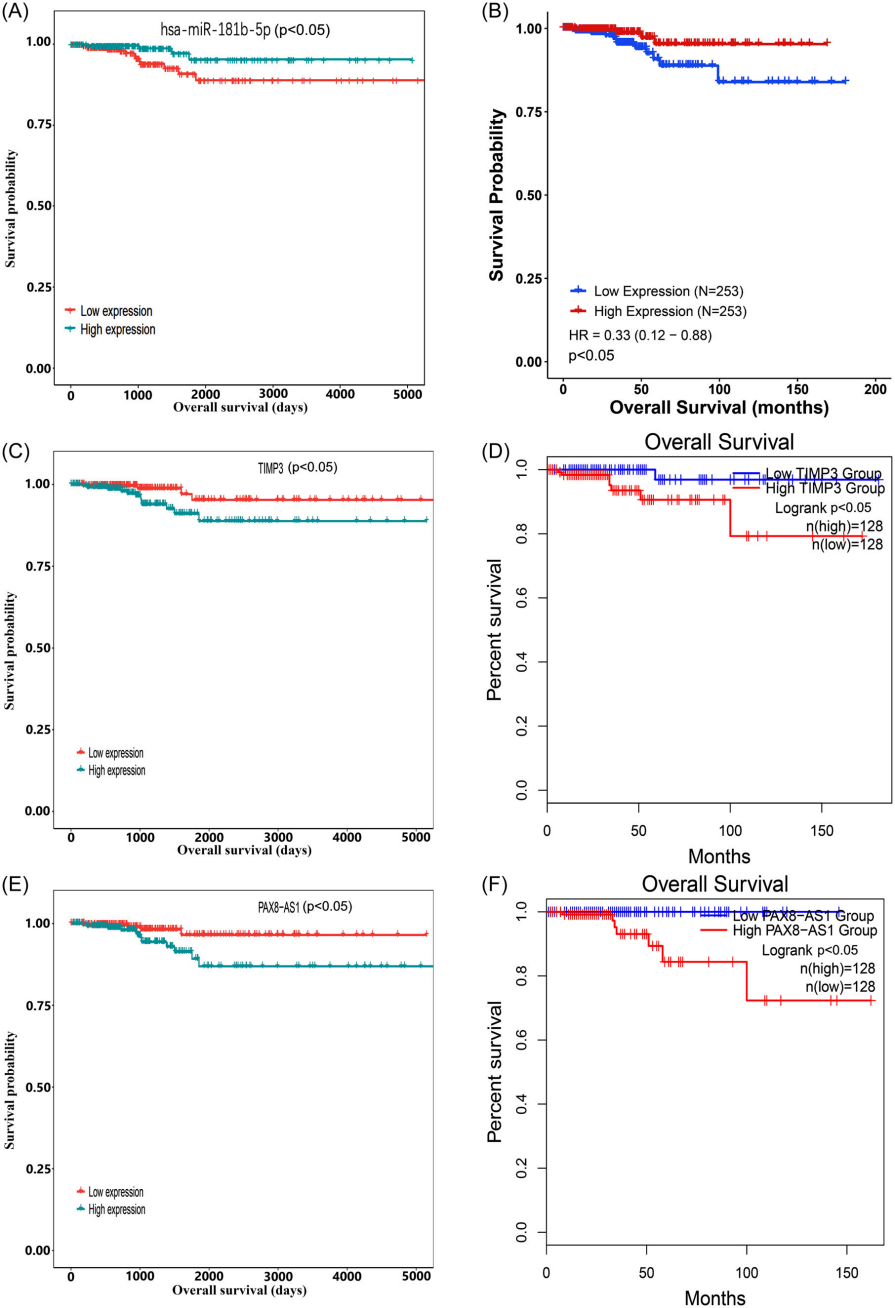
Overall survival analysis of RNAs. (A) and (B) Survival analysis of has‐miR‐181b‐5p. (C) and (D) Survival analysis of TIMP3. (E) and (F) Survival analysis of PAX8‐AS1. HR, hazard ratio.

To identify potential deRNAs with prognostic value, a survival package was employed to assess their impact on survival. Our analysis revealed that 161 mRNAs, nine miRNAs, and 14 lncRNAs were significantly correlated with the overall survival rate (*p* < 0.05). Further examination of the competing endogenous RNA (ceRNA) network identified hsa‐miR‐181b‐5p as the central node in the network and significantly associated with survival (*p* < 0.05). Remarkably, patients with high expression of hsa‐miR‐181b‐5p exhibited a distinct difference in overall survival rates compared to those with low expression (Figure 5A), and this result was corroborated by the findings obtained from CancerMIRNome (Figure 5B). Consequently, hsa‐miR‐181b‐5p was selected as the hub node to construct the ceRNA axis related to the prognosis of TC.

### 3.5 Construction of ceRNA axis related to the prognosis of TC

**Figure 6:**
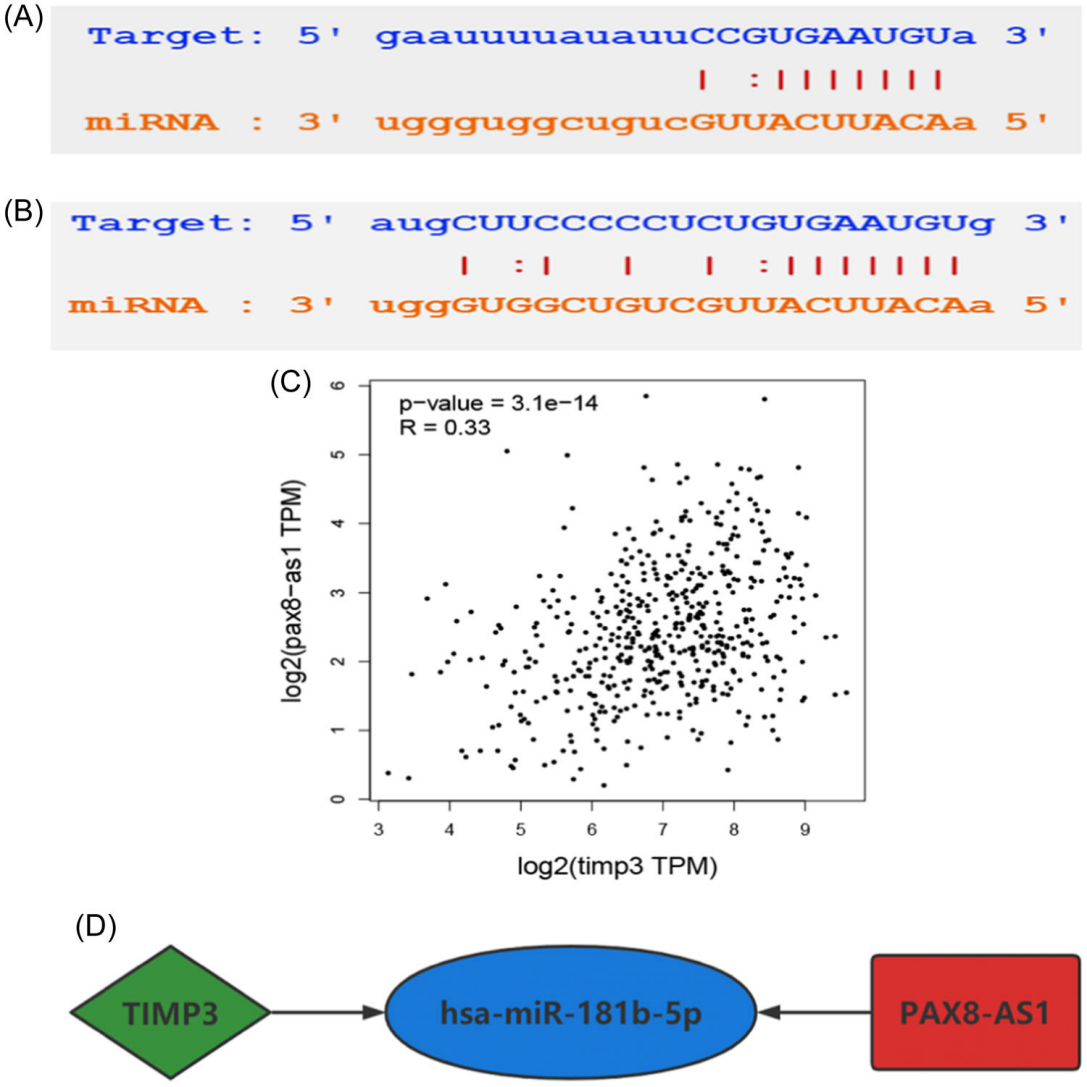
(A) and (B) The binding sites of hsa‐miR‐181b‐5p with TIMP3 and PAX8‐AS1. (C) Correlation analysis of TIMP3 and PAX8‐AS1. (D) ceRNA axis of the network.

Utilizing the Targetscan tool, we have found that hsa‐miR‐181b‐5p has the ability to target and regulate 14 ceRNAs, namely BCL2, DDIT4, DRAM1, DUSP5, FOS, PLAG1, SCD, SLC25A25, SPP1, TIMP3, TNFRSF11B, ZFP36L1, ZFP36L2, and ZNF781. Moreover, it is a potential regulator of 7 lncRNAs, including ST7‐AS1, LIFR‐AS1, AL158206.1, PAX8‐AS1, AL138756.1, AC124312.3, LINC01806. Among the above genes, TIMP3 (*p* < 0.05) and PAX8‐AS1 (*p* < 0.05) were significantly correlated with the overall survival rate, with individuals expressing high levels of these genes having a poor prognosis. This conclusion was further supported by analysis using the GEPIA2 database (Figure 5C–F). Then, the binding sites of hsa‐miR‐181b‐5p, TIMP3, and PAX8‐AS1 were retrieved through Starbase to validate their targeting relationships (Figure 6A,B). PAX8‐AS1 and TIMP3 had the same detrimental impact on the prognosis of patients with TC, in contrast to the effect observed with hsa‐miR‐181b‐5p. The ceRNA hypothesis assumes that lncRNA can competitively bind to miRNA, resulting in a reduction in targeted miRNA and an increase in mRNA expression. Hence, it was hypothesized that PAX8‐AS1 can bind to hsa‐miR‐181b‐5p and indirectly promote the expression of TIMP3.

According to the aforementioned hypothesis, lncRNA in the ceRNA network is positively correlated with mRNA, so it is anticipated that TIMP3 and PAX8‐AS1 would demonstrate a positive correlation. To verify this surmise, the GEPIA2 database was used, which revealed that PAX8‐AS1 could be used as a ceRNA to promote the expression of TIMP3 by competitively binding to hsa‐miR‐181b‐5p (Figure 6C). Consequently, a ceRNA axis TIMP3/hsa‐miR‐181b‐5p/PAX8‐AS1 was constructed, which was found to be closely related to the prognosis of TC (Figure 6D).

Moreover, an investigation was conducted to explore the relationship between gene expression (TIMP3, hsa‐miR‐181b‐5p, and PAX8‐AS1) and clinicopathological features. The results showed a significant association between TIMP3 expression and AJCC N stage, while no significant correlations were found with age, sex, AJCC T stage, AJCC M stage, and clinical stage. PAX8‐AS1 expression exhibited a significant correlation with AJCC N stage and clinical stage, but no significant associations were observed with age, sex, AJCC T stage, and AJCC M stage. The expression of hsa‐miR‐181b‐5p was significantly associated with age, but no significant associations were observed with AJCC T, AJCC N, AJCC M, and clinical stage (Table 2).

**Table 2:** Correlation between gene expression (TIMP3, hsa‐miR‐181b‐5p and PAX8‐AS1) and clinicopathological variables.

Clinicopathological features	Cases	TIMP3			PAX8‐AS1			hsa‐miR‐181b‐5p		
		High	Low	*p*	High	Low	*p*	High	Low	*p*
Age	500	250	250		250	250		250	250	
Old (>60)	120	67	53	>0.05	58	62	>0.05	49	71	<0.05
Young (<60)	380	183	197		192	188		201	179	
Gender	500									
Female	365	190	175	>0.05	182	183	>0.05	180	185	>0.05
Male	135	60	75		68	67		70	65	
T	498	249	249		248	250		249	249	
T1	142	83	59	>0.05	78	64	>0.05	76	66	>0.05
T2	164	80	84		88	76		88	76	
T3	169	78	91		73	96		73	96	
T4	23	8	15		9	14		12	11	
N	450	220	230		220	230		221	229	
N0	228	124	104	<0.05	125	103	<0.05	107	121	>0.05
N1	222	96	126		95	127		114	108	
M	289	135	154		150	139		142	147	
M0	280	133	147	>0.05	146	134	>0.05	136	144	>0.05
M1	9	2	7		4	5		6	3	
Stage	498	249	249		248	250		248	250	
Stage I	279	139	140	>0.05	151	128	<0.05	149	130	>0.05
Stage II	52	31	21		30	22		24	28	
Stage III	112	54	58		47	65		48	64	
Stage IV	55	25	30		20	35		27	28	

### 3.6 Intracellular localization of PAX8‐AS1

**Figure 7:**
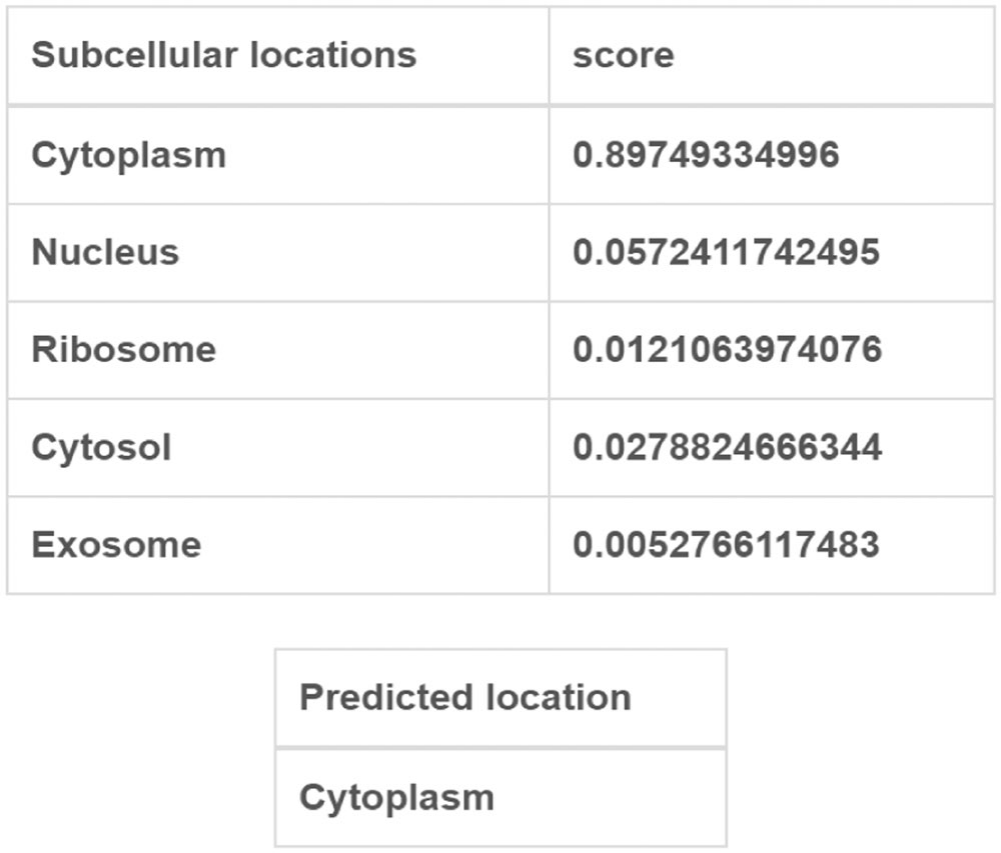
Subcellular localization of PAX8‐AS1.

The subcellular localization of lncRNA is crucial for its potential functions. When located in the nucleus, lncRNA regulates the transcription process through chromatin interaction and remodeling. Conversely, lncRNA found in the cytoplasm is involved in mediating signal transduction pathway, translation procedure, and posttranscriptional control of gene expression.^30^ Furthermore, the endogenous competition of lncRNA mainly occurs in the cytoplasm. To clarify the role of PAX8‐AS1, we obtained the sequence information of PAX8‐AS1 from the UCSC website and conducted a subcellular localization analysis using lncLocator. Our analysis discovered that PAX8‐AS1 predominantly exists in the cytoplasm (Figure 7). These findings, once again, indicated that PAX8‐AS1 may act as a ceRNA that regulates the expression of TIMP3 by competitively binding to hsa‐miR‐181b‐5p.

### 3.7 Functional enrichment analysis of TIMP3

**Figure 8:**
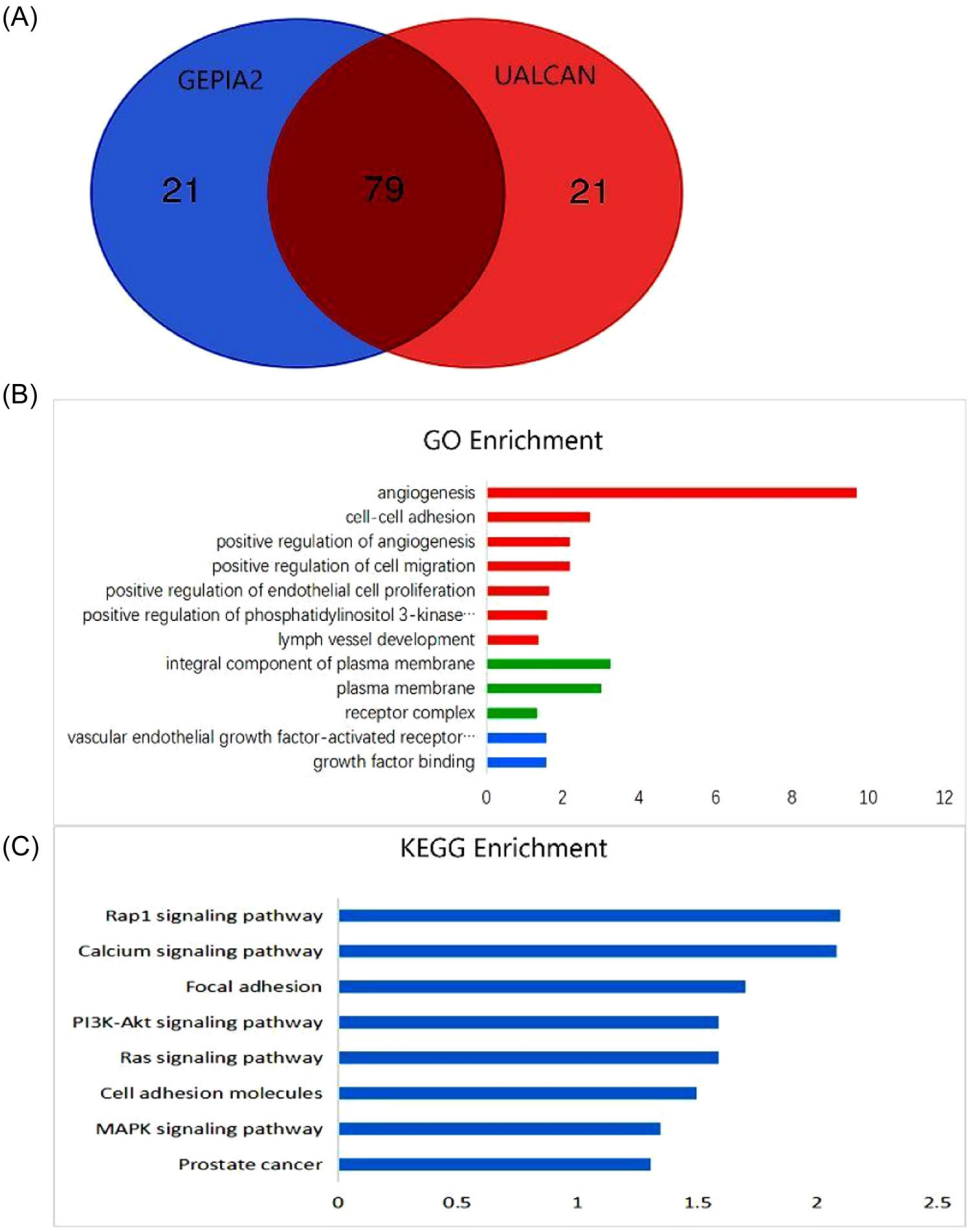
Analysis of biological function of TIMP3. (A) Venn diagram of TIMP3 coexpression genes. (B) GO enrichment analysis of TIMP3. (C) KEGG enrichment analysis of TIMP3. GO, gene ontology; KEGG, Kyoto Encyclopedia of Genes and Genomes; MAPK, mitogen‐activated protein kinase.

To understand the function of TIMP3, we employed the UALCAN and GEPIA2 databases to identify the top 100 genes that display coexpression with TIMP3. Our analysis found that 79 genes were consistently identified in both databases (Figure 8A). Subsequently, we examined the functions of these genes using the DAVID database to infer the biological functions of TIMP3. GO enrichment analysis demonstrated that the genes coexpressing with TIMP3 were primarily involved in various biological processes, including angiogenesis, cell adhesion, and cell migration. Moreover, these genes were found to be involved in molecular functions such as vascular endothelial growth factor‐activated receptor activity and growth factor binding. Furthermore, the analysis revealed that these genes contribute to the formation of cellular components, such as the composition of the plasma membrane and receptor complex (Figure 8B). Besides, the KEGG pathway analysis indicated that TIMP3 was principally implicated in Rap1 pathway, calcium signaling pathway, focal adhesion, PI3K‐Akt, and other signaling pathways (Figure 8C). These findings suggest that TIMP3 may be involved in the mechanisms of cell adhesion and migration by modulating plasma membrane and receptor complexes, thus affecting the progression of TC.

### 3.8 Correlation between TIMP3 expression and immunity of TC

Previous studies have shown that certain adhesion molecules can act as immunoglobulins to promote the invasion and migration of tumor cells and ultimately induce tumor immune evasion, which is closely linked to tumor progression and poor prognosis.^31^ Given the biological function of TIMP3, it is of great significance to study its correlation with the immune response in TC. Hence, we examined the relationship between TIMP3 and TC immunity, specifically focusing on tumor immune cell infiltration, immunity regulators, chemokines, and their receptors.

**Figure 9:**
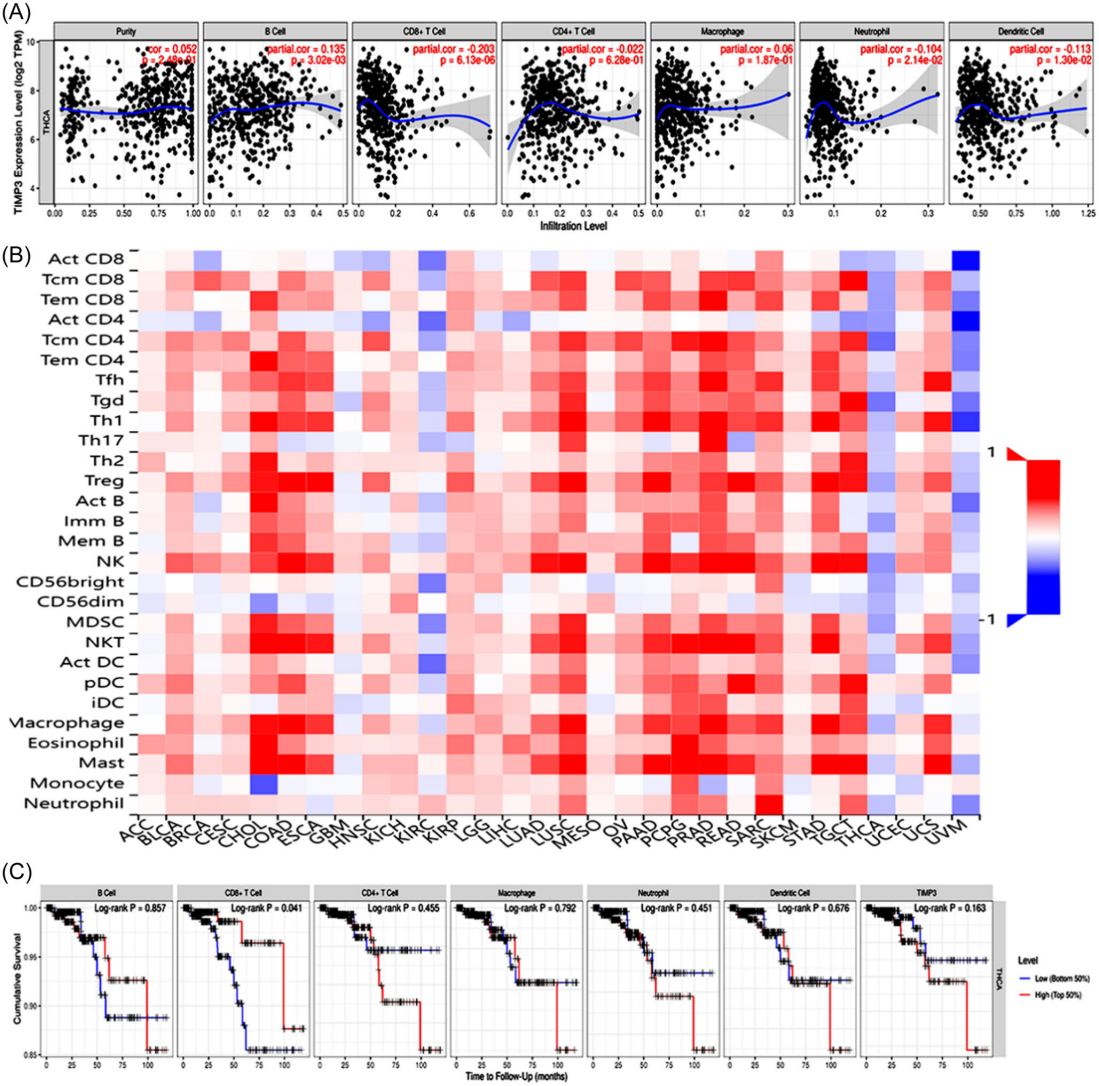
Association between TIMP3 and immune cells. (A) and (B) Relationship between TIMP3 and immune cells based on the data of TIMER and TISIDB database. (C) The relationship between immune cells and prognosis of TC. TC, thyroid cancer.

To explore the relationship between TIMP3 and immune cells in TC, we conducted a comprehensive search in both the TIMER and TISIDB databases. The analysis of the TIMER database showed a positive correlation between TIMP3 expression and the infiltration level of B cells. Contrastly, TIMP3 expression was found to be negatively correlated with the infiltration level of CD8+ T cells, neutrophils, and dendritic cells, but showed no relationship with CD4+ T cells and macrophages (Figure 9A). On the other hand, the results of TISIDB database revealed a negative correlation between TIMP3 expression and the presence of ActCD4 cells, macrophages, ActDC cells, ActCD8 cells, neutrophils, and ActB cells (Figure 9B). The search results from the two databases suggest that TIMP3 may affect the immune infiltration of TC by primarily decreasing the infiltration abundance of CD8+ T cells, neutrophils, and dendritic cells in the immune microenvironment. In addition, the analysis conducted using the TIMER database exhibited that only CD8+ T cells were significantly related to the prognosis of TC. Specifically, patients with high levels of CD8+ T cell infiltration had a more favorable prognosis (Figure 9C). The above‐mentioned findings suggest that TIMP3 might lead to an unfavorable prognosis in patients with TC by inhibiting the infiltration of CD8+ T cells in the tumor microenvironment.

**Figure 10:**
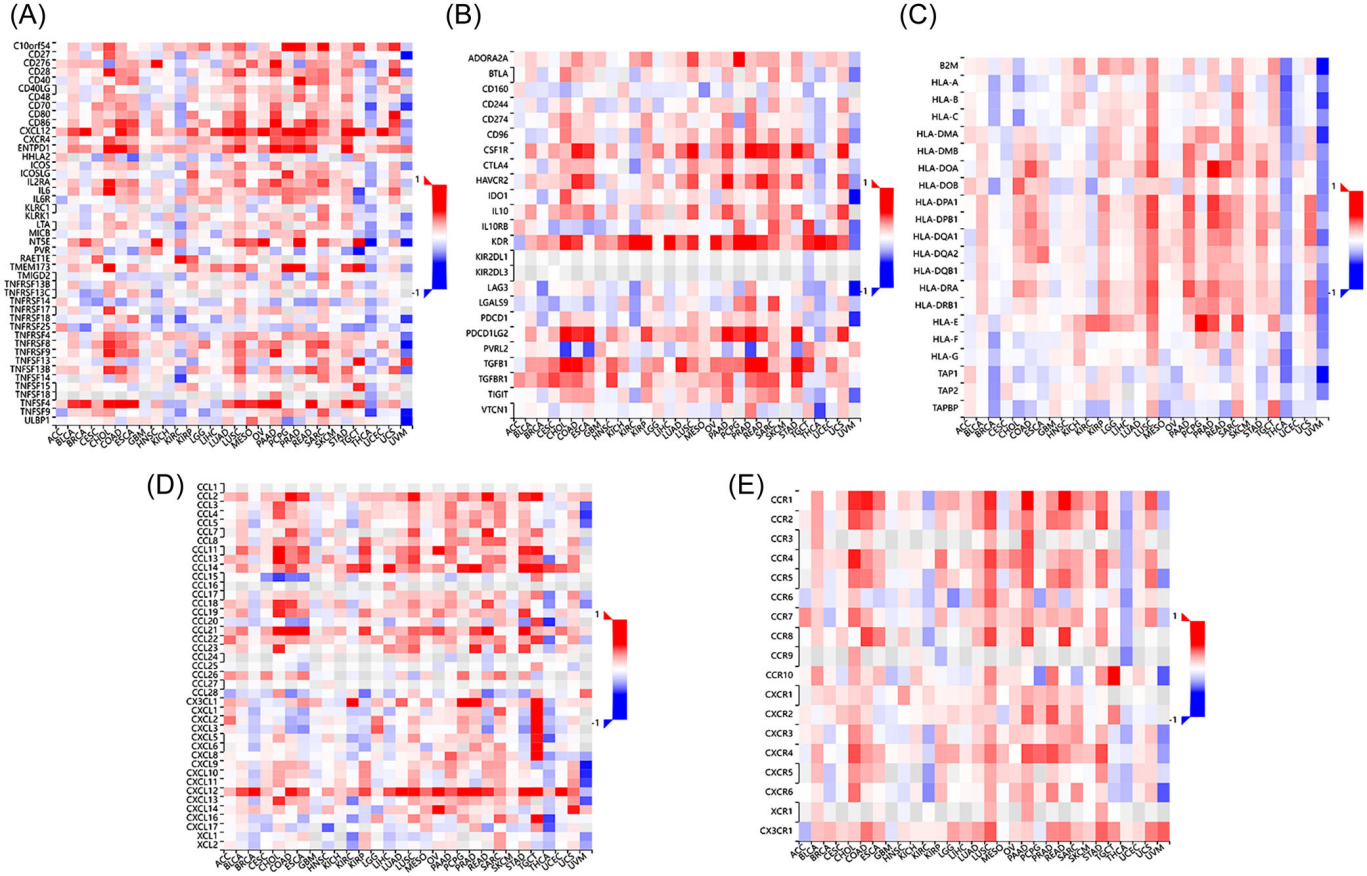
Correlation analysis of TIMP3 expression and immune factors. (A)–(E) Association between TIMP3 expression and immunostimulators, immunosuppressive factors, major histocompatibility complex molecules, chemokines, and chemokine receptors.

To further understand the impact of TIMP3 on tumor immune response, an analysis was conducted using the TISIDB database. This analysis aimed to examine the correlation between TIMP3 expression and the expression of immunomodulators, which encompass immunostimulators, immunosuppressive factors, and MHC molecules. The results of this analysis showed that TIMP3 was negatively correlated with the majority of immunostimulators, immunosuppressive factors, and all MHC molecules. In addition, an important positive correlation was observed between the expression levels of CXCL12, ENTPD1 (immunostimulators), KDR, ADORA2A, PVRL2 (immunosuppressive factors), as well as TIMP3 (Figure 10). This indicates that TIMP3 might play a regulatory role in the immune response of tumor cells by coordinating the expression of these immunomodulators.

Chemokines and their receptors, as mediators of immune cell transport, participate in all stages of tumor development, play an important role in the formation of the tumor microenvironment, and exhibit dual functions by both promoting and inhibiting tumors.^32^ To investigate the relationship between TIMP3 and chemokines, as well as their receptors, a study was conducted using the TISIDB database. The findings of this study revealed that TIMP3 expression was negatively correlated with the expression of most chemokines and their receptors, but positively associated with the expression of CCL14, CXCL12, and CCL21 (Figure 10). These results suggest that TIMP3 might negatively regulate the expression of chemokines and their receptors, thereby affecting the transportation of immune cells in TC.

## 4 DISCUSSION

The incidence of TC, a common malignant tumor, has been steadily rising.^1^ Although most of them progress slowly and have a benign course, there are certain types that are resistant to standard treatment, have a tendency to recur and metastasize, and ultimately have a poor prognosis.^2^, ^3^ Therefore, it is crucial to explore the underlying mechanisms involved in the development and progression of TC and identify new biomarkers for treating refractory TC and improving its prognosis.

In recent years, mounting evidence has shown that the ceRNA network is involved in the development and progression of various types of cancer, such as gastric cancer, pancreatic cancer, breast cancer, and presumably TC.^33^, ^34^, ^35^, ^36^ For example, a study by Zhang et al. revealed that the regulatory network of TGFB1, hsa‐mir‐429/hsa‐mir‐199a‐3p, and their targeted genes might be an important contributor to the development of medullary thyroid carcinoma.^37^ Another study proposed that Klhl14‐AS could affect the differentiation of thyroid cells and the proliferation of cancer cells by binding to Mir182‐5p and Mir20a‐5p.^38^ Additionally, Chu et al. found that circRUNX1 might promote the progression and metastasis of thyroid carcinoma through its interaction with MiR‐296‐3p and subsequent regulation of DHD2 expression.^39^ However, despite these advancements that have been made in the research of ceRNA networks related to TC, the exact mechanisms by which ceRNA networks participate in the pathogenesis of TC have not been fully clarified.

To find out the potential role of additional regulatory networks in determining the prognosis of TC prognosis, we analyzed RNA samples from TC tissues and normal tissues obtained from the TCGA database. We identified deRNAs and constructed a ceRNA network using various databases such as miRcode, TargetScan, miRTarbase, and Starbase. Moreover, we conducted a survival analysis to evaluate the prognostic significance of nodes within the network. Our findings showed that TC patients who had elevated levels of hsa‐miR‐181b‐5p or decreased levels of TIMP3 or PAX8‐AS1 had a more favorable prognosis. Based on these findings, we have identified the ceRNA axis involving TIMP3/hsa‐miR‐181b‐5p/PAX8‐AS1 as being related to the prognosis of TC.

The research findings presented in this study indicate that hsa‐miR‐181b‐5p plays a central role in this ceRNA network and is significantly correlated with the survival of TC patients. Previous studies have reported the involvement of hsa‐miR‐181b‐5p in the development, progression, and prognosis of various malignancies. For instance, Yuan et al. found that decreased expression of hsa‐miR‐181b‐5p in osteosarcoma cells significantly impair the antitumor activity of ALKBH5.^40^ Similarly, Hu et al. reported that ST7‐AS1 promotes the malignant transformation of lung adenocarcinoma cells by regulating the hsa‐miR‐181b‐5p/KPNA4 axis.^41^ Additionally, Di Marco et al. demonstrated that the clinical progression of B‐cell chronic lymphocytic leukemia (CLL) is related to the substantial decrease of hsa‐miR‐181b‐5p, which can promote the death of CLL cells by regulating immune function.^42^ Furthermore, several studies have observed that hsa‐miR‐181b‐5p plays an important role in regulating various cellular processes, including cell proliferation, differentiation, apoptosis, immune inflammatory response, and anticancer drug sensitivity.^43^, ^44^, ^45^, ^46^, ^47^ Nevertheless, the involvement of hsa‐miR‐181b‐5p in the development, progression, and prognosis of TC has not yet been studied. This study aimed to investigate the correlation between hsa‐miR‐181b‐5p expression and the survival of TC patients. The results revealed a significant association between high expression of hsa‐miR‐181b‐5p and improved prognosis in TC patients. Based on these observations, it is speculated that the upregulation of hsa‐miR‐181b‐5p may hinder TC progression and enhance patient prognosis. Additionally, this study assumed that hsa‐miR‐181b‐5p may affect the prognosis of TC by negatively regulating TIMP3 and PAX8‐AS1. However, further validation through in vitro and in vivo experiments is necessary to elucidate the precise nature of the exact relationship between these factors.

TIMP3, a matrix metalloproteinase inhibitor involved in the degradation of the ECM, is a target gene regulated by hsa‐miR‐181b‐5p and has been associated with cell mitosis.^47^ It has been proven to be involved in the proliferation, invasion, and metastasis of various cancers, such as nonsmall cell lung cancer, melanoma, gastric cancer, and prostate cancer.^48^, ^49^, ^50^, ^51^ Studies have also shown that TIMP3 might work as a tumor suppressor in TC by inhibiting angiogenesis and macrophage infiltration.^52^ These findings align with our findings that highlight the involvement of TIMP3 in cellular processes including angiogenesis, cell adhesion, and cell migration. In recent years, the role of cell adhesion and migration in cancer metastasis and immunity responses has been increasingly recognized.^53^, ^54^ Some studies have shown that TC cells could escape immune responses by suppressing immune the microenvironment.^55^, ^56^ To better understand the molecular and immunological characteristics of the tumor microenvironment, this study further explored the relationship between TIMP3 and the immune microenvironment in TC. The results revealed a negative correlation between TIMP3 and the abundance of CD8+ T cells, as well as the expression levels of most immunosuppressive factors, immunostimulatory factors, MHC molecules, most chemokines, and their receptors in the immune microenvironment. Moreover, CD8+ T cells have been identified as crucial effector cells in antitumor immunity, and their high level of infiltration in TC was found to be associated with a more favorable prognosis. Conversely, higher expression of TIMP3 was linked to a poorer prognosis, which suggests that TIMP3 might lead to unfavorable clinical outcomes in TC patients by decreasing the abundance of infiltrated CD8+ T cells. In addition, prior studies have shown that CD8+ T cells produce CXCL9/10/11 chemokines, which possess antitumor effect.^57^ The present study revealed a negative correlation between TIMP3 and CXCL9/10/11, providing further support for the findings regarding immune infiltration and offering a partial explanation for the association between elevated expression of TIMP3 and unfavorable prognosis. Considering the dual effects of immunomodulatory factors, chemokines, and their receptors on tumor progression,^32^, ^58^, ^59^ additional in vitro and in vivo studies are necessary to investigate the underlying mechanism linking TIMP3 to the immune environment and clinical prognosis.

PAX8‐AS1 is an lncRNA regulated by hsa‐miR‐181b‐5p. The GEPIA2 database has confirmed that PAX8‐AS1 can act as a ceRNA by competitively binding to hsa‐miR‐181b‐5p and thereby promoting the expression of TIMP3. PAX8‐AS1 is an upstream gene of PAX8, a transcription factor of the PAX family, which plays an important role in the organogenesis of female thyroid, kidney, and Muller system.^60^ Previous studies have suggested that PAX8 may contribute to the carcinogenesis by acting as an inhibitor of cell differentiation and apoptosis.^61^ Similarly, PAX8‐AS1, located at the upstream of the PAX8 gene, has been implicated in tumor progression by positively regulating PAX8 expression.^62^ Moreover, PAX8‐AS1 has been reported to be involved in the development and progression of various diseases, including juvenile acute lymphoblastic leukemia, cervical cancer, gestational diabetes, and triple‐negative breast cancer.^62^, ^63^ However, limited information is available regarding the association between PAX8‐AS1 and the development and progression of TC. This study found that TC patients with high PAX8‐AS1 expression had a poor prognosis, in line with previous research demonstrating shorter RFS in TC patients with high PAX8‐AS1 expression.^64^ These findings suggest that PAX8‐AS1 could be a potential therapeutic target for improving the prognosis of TC patients.

## 5 CONCLUSION

In this study, a comprehensive analysis of the interactions between deRNAs in TC has been conducted, resulting in the identification of a potential axis involving TIMP3/hsa‐miR‐181b‐5p/PAX8‐AS1. This axis has been found to be closely associated with the prognosis of TC. The study has also revealed the role of the TIMP3/hsa‐miR‐181b‐5p/PAX8‐AS1 axis in the development and progression of TC, particularly in its regulation of infiltrating immune cells, immune regulators, and chemokines, which ultimately impacts the clinical prognosis of patients. These findings suggest that targeting the TIMP3/hsa‐miR‐181b‐5p/PAX8‐AS1 axis may be a potential therapeutic strategy for refractory TC, with the aim of improving its prognosis.

However, it is important to acknowledge certain limitations in this study. First, the reliance on data solely from the TCGA database may introduce potential biases and compromise the reliability of the conclusions. Second, the absence of in vitro and in vivo experiments is a limitation in confirming the targeting relationship of the TIMP3/hsa‐miR‐181b‐5p/PAX8‐AS1 axis in TC. Lastly, the study did not conduct functional tests, which are essential for investigating biological function, underlying mechanism, and the specific relationship with tumor immunity.

## AUTHOR CONTRIBUTIONS

**Jiamin Liang**: Conceptualization; resources; writing—original draft; writing—review and editing. **Yu Deng**: Data curation; validation. **Yubi Zhang**: Data curation; formal analysis; validation; visualization. **Bin Wu**: Funding acquisition; methodology; supervision; writing—review and editing. **Jing Zhou**: Funding acquisition; methodology; project administration; supervision; writing—review and editing.

## CONFLICT OF INTEREST STATEMENT

The authors declare no conflict of interest.

## TRANSPARENCY STATEMENT

The lead author Bin Wu, Jing Zhou affirms that this manuscript is an honest, accurate, and transparent account of the study being reported; that no important aspects of the study have been omitted; and that any discrepancies from the study as planned (and, if relevant, registered) have been explained.

## Data Availability

All data generated or analyzed during the current study are publicly available in the TCGA database (https://portal.gdc.cancer.gov/), and the dbGaP Study Accession number is phs000178.
